# Role of ambulatory blood pressure monitoring in elderly hypertensive patients

**DOI:** 10.1186/s40885-022-00205-6

**Published:** 2022-07-01

**Authors:** Miguel Camafort, Wook-Jin Chung, Jin-Ho Shin

**Affiliations:** 1 ESH Excellence Hypertension Center, Department of Internal Medicine, Geriatrics Section, Hospital Clinic, University of Barcelona, Barcelona, Spain; 2 Research Group on Cardiovascular Risk, Nutrition and Aging, August Pi i Sunyer Biomedical Research Institute (IDIBAPS), Barcelona, Spain; 3 Research Group CB06/03/0019, Biomedical Network Research Center in Obesity and Nutrition (CIBER-OBN), Institute of Health Carlos III (ISCIII), Ministry of Science, Innovation and Universities, Madrid, Spain; 4 Department of Cardiovascular Medicine, Gachon University Gil Medical Center, Incheon, Republic of Korea; 5 Gachon Cardiovascular Research Institute, Gachon University, Incheon, Republic of Korea; 6 Division of Cardiology, Department of Internal Medicine, Hanyang University College of Medicine, Seoul, Republic of Korea

**Keywords:** Arterial hypertension, Aging, Elderly, Out of office blood pressure measurements, Ambulatory blood pressure monitoring, White coat hypertension, Blood pressure variability, Nocturnal hypertension

## Abstract

**Background:**

Arterial hypertension is facing some changes in the last years. Its prevalence is increasing in elderly subjects. This growing prevalence is due to longer survival of the population worldwide, among other factors. On the other hand, recent guidelines have insisted in the relevance of out of office blood pressure measurements, to improve diagnostic and management of hypertension. Therefore, elderly subjects with hypertension could benefit from out of office blood pressure measurements, like ambulatory blood pressure measurements; nevertheless, there are very few or no specific recommendations regarding this.

**Aim:**

In this review, we will gather the most important information about this subject.

**Results:**

As hypertension in the elderly has some specific characteristics related to aging of the cardiovascular system, the most important aspect could be that these characteristics make ambulatory blood pressure measurement suitable for its use in elderly. Among those a higher prevalence of white coat hypertension, white coat phenomenon, and a higher nocturnal blood pressure and higher prevalence of nondipper and riser pattern, represent aspects that should be considered for better diagnostic and an improved management.

**Conclusion:**

As the prevalence of hypertension will grow in the next years, more studies specifically directed to this subject are needed.

**Supplementary Information:**

The online version contains supplementary material available at 10.1186/s40885-022-00205-6.

## Background

Arterial hypertension is the principal risk factor for cardiovascular disease (CVD) and affects a portion of the population equivalent to 22.3% globally and 26.5% in middle income countries [1]. Hypertension is a global pandemic that is growing fast. In 2010, some estimates suggested that one in three of adults, worldwide, had hypertension [2]. This prevalence is growing; throughout the last years, the number of adults with raised blood pressure increased from 594 million in 1975 to 1,13 billion in 2015 [3]. In Korea, recent data show, that in females the estimated number of people with hypertension is 1.96 million for men and 2.99 million for women among the population aged 65 or older [4]. This increase is due to different factors, one of them being longer survival of the population worldwide, making hypertension more prevalent in older adults [5].

Another important aspect related to hypertension is that all recent guidelines have highlighted the relevance of out of office blood pressure (BP) measurements in diagnosis, evaluation, and management of hypertension, mainly ambulatory BP measurement (ABPM) and home blood pressure measurement [6–13]. This is mainly due to some aspects of ABPM, which make it, more suitable than office BP measurement (OBPM) for diagnosis and management. In summary ABPM gives a larger number of readings than OBPM, provides a profile of BP behavior in the patient’s usual daily environment, allows identification of white coat hypertension phenomena (defined as refers to the condition in which BP is elevated in the office, but is normal when measured by ABPM, HBPM, or bot h[8])and masked hypertension phenomena (defined as refers to the condition in which in whom the BP is normal in the office, but is elevated when measured by HBPM or ABP M[8]), demonstrates nocturnal hypertension (defined as Night-time (or asleep) mean SBP > _120 and/or mean DBP > _70 [8]), assesses dipping pattern (defining dipper if their nocturnal BP falls by > 10% of the daytime average BP value [8]), and BP variability over the 24-h period, assesses the 24-h efficacy of antihypertensive medication, and is a stronger predictor of cardiovascular morbidity and mortality than OBPM [6]. Therefore, in this narrative review we aimed to search for recommendations advantages and particularities of ABPM in elderly patients.

### Current recommendations for ABPM in elderly

But what do clinical guidelines, and position papers, recommend on ABPM in elderly subjects? There is no specific guideline for this subject. In fact, the 2021 European Society of Hypertension practice guidelines for office and out of office BP measurement [14] define the clinical indications for ABPM with no specific reference to elderly. Nevertheless, the previous European Society of Hypertension Position Paper on ABPM, published in 2013 [6] includes assessing hypertension in the elderly in the clinical indications for ABPM, since some major age-related changes could only be ascertained through ABPM [15, 16]:

- White coat hypertension (WCH): That is more common in older than in younger adults.
- Pulse pressure: As there is a predominant increase in 24-h systolic BP with a mild decrease in 24-h diastolic BP after 60–70 years of age, as a result, an increase in 24-h pulse pressure occurs and, therefore, the prevalence of 24-h isolated systolic hypertension is higher.
- Nondipping pattern is more common in the elderly.
- Exaggerated BP variability and morning BP surge is also more common in elderly subjects.
- Postural and postprandial hypotension is also more common.

Other guidelines point out that out of office BP, preferably ABPM, in addition to an accurate, standardized OBPM, should be undertaken to exclude white coat effect [12, 13].

These changes are, often, indicative of autonomic failure, therefore it is important to identify them, so that treatment can be tailored to take account of such fluctuations in BP. They are, also, relevant as outcome studies in the elderly have shown that 24-h systolic BP, as 24-h ambulatory pulse pressure are more closely associated with cardiovascular events, cardiovascular mortality, and total mortality than office BP, being these associations closer for stroke than coronary artery disease [15–20].

More specifically, the Spanish ABPM Guidelines recommend ABPM in elderly patients since this population group has specific characteristics that requires an ABPM to confirm, such as a higher prevalence of WCH, an alteration of the circadian profile with a higher proportion of the reduced dipping profile and a higher prevalence of postprandial and orthostatic hypotension (OH). All these alterations are associated with increased cardiovascular risk and mortality. Poor sleep quality in the elderly can affect the interpretation of ABPM results [21].

### Specifical characteristics of hypertension in the elderly in which ABPM could be helpful

Hypertension in elderly subjects is a special situation due to different aspects. We know that the prevalence of hypertension rises with age, particularly, in Korea among hypertensive patients, the proportion of hypertensive patients aged 65 years or older was 37.4% in 2018 [14]. A recent systematic review, including 135 population-based studies of near a million adults from 90 countries found that, in adults aged ≥70 years, the estimated prevalence of hypertension was 73.6% for men and 77.5% for women in high income countries and 65.6% for men and 74.7% for women in low- and middle-income countries [5].

Beside the increasing prevalence [22], there are other characteristics of hypertension in elderly subjects. As for the phenotype of hypertension in the elderly some differential characteristics have been established.

### Isolated systolic hypertension

Cross-sectional and longitudinal population studies have shown that systolic BP continuously increases with age, while diastolic BP rises until 50 years of age and then levels off or even slightly decreases [23]. This phenomenon, together with the arterial stiffening occurring in all great arteries, explains why the prevalence of isolated systolic hypertension rises with age and becomes the predominant type of hypertension in both treated and untreated elderly subjects, a highly significant problem in elder subjects. These changes cause an increase in pulse pressure which is known as a risk factor for clinical and subclinical cardiovascular disease [24, 25]. Besides that, some data point out that a lower diastolic BP could cause greater morality in older hypertensive patients [25].

### White coat hypertension

It is also called isolated office hypertension, and it is referred to those patients that present a persistent BP elevation in the office with a normal BP outside of the office. This phenomenon is more prevalent in older subjects, and advancing age is the strong determinant of WCH [26]. In the hypertension in the very elderly trial (HYVET), white coat effect, defined as clinic BP minus daytime ambulatory BP, were about 40 mmHg and 20 mmHg in systolic BPs at baseline and follow-up, respectively [27].

### Nocturnal hypertension and nondipping pattern

In older hypertensive patients, one of the age-associated BP abnormalities could be the loss of nocturnal BP dip, therefore, the proportion of nondippers tends to increase compared with that of in younger subjects [26]; in some studies, up to 43% of elderly hypertensive patients could have a nocturnal BP elevation [28], this is even more frequent in elderly frail subjects [29]. These changes in circadian pattern, among elderly hypertensive patients, are associated with an increased cardiovascular risk. So, uncontrolled nocturnal hypertension is a predictor of future cardiovascular events [30]. Nevertheless, other studies have found some differences between treated and untreated elderly patients, in reference to high CV risk pattern. So, in elderly hypertensive treated patient, the extreme dipper is associated to a high morning surge, followed by the reverse dippers, nondippers, and dippers with a high morning surge. But, in general elderly population, the highest risk pattern was observed among the reverse dippers, followed by the nondippers, extreme dippers with a high morning surge, and dippers with a high morning surge [31].

However, we should not forget that quality of sleep-in elderly patients can be affected by concomitant conditions, such as prostatic hypertrophy, sleep apnoea, and sleep fragmentation, and in such cases, nocturnal BP loses some of its prognostic significance [5].

### Orthostatic hypotension

This type of BP dysregulation could be very frequent in elderly. In community dwelling, Asian subjects, the prevalence of OH was found to be an 11.0% [32]. However, in a recent meta-analysis, a 35.2% of geriatric outpatients had OH [33]. Moreover, one recent post-hoc analysis of a subpopulation in the SPRINT trial examined the association of OH with WCH and systolic BP night reduction. Those with OH, had higher prevalence of WCH (15% vs. 7% without OH) and OH was significantly associated (odds ratio [OR], 2.24; 95% confidence interval [CI], 1.28–4.27). Those with OH also have a higher prevalence of nondipping pattern (25% vs. 14% without OH) being significantly associated to reverse dipping (OR, 2.29; 95% CI, 1.31–3.99) [34].

Vallelonga et al. have found that some selected ABPM parameters (number of hypotensive episodes and the presence of awakening hypotension), may be used to screen patients for OH [35].

### Other aspects related to ABPM in elderly

Beside these phenotypic characteristics of hypertension in elderly subjects, this population presents some other relevant problems. One of them is that the available evidence on the efficacy and safety of BP lowering treatment in these patients is scarce [36].

#### Frailty

Another important question to be considered is the possibility of an association between hypertension and frailty syndrome in older patients. This possibility is far from clear for various reasons as, for example, the different definitions and tools used to measure of frailty in the available studies, that are, by the way, very few and with low number of subjects included.

As for the influence of frailty on the 24-h BP pattern, it has been explored in a short study with population above 80 years. In this study, frail patients had higher nocturnal systolic BP, and were independently associated with higher risk of nondipping and reduced nighttime systolic BP fall [29].

In another retrospective study, Zhu et al. [37] included elderly patients with essential hypertension who underwent ABPM, assessing frailty by a Rockwood’s frailty index. A greater blood systolic BP variability (particularly average real variability [ARV] and coefficient of Variability) were independently associated with higher frailty status.

#### Cognitive decline

Cognitive decline is increasing its prevalence and it is known that a well-designed management of hypertension, starting on middle age may reduce the onset of dementia in the elderly [38].

Kim et al. [39] in a recent paper investigated whether higher BP variability (BPV) is associated with faster declines in cognitive function in 1240 Korean ischemic stroke patients, belonging to the PICASSO trial. After adjusting for multiple variables, higher BPV was independently associated with faster cognitive decline over time, measured by Mini-Mental State Examination (MMSE) and Montreal Cognitive Assessment, with no significant intergroup difference in cognitive changes associated with mean systolic BP.

Another Chinese study was aimed at identifying the relevance of ABPM in cognitive disfunction assessed through MMSE and magnetic resonance imaging [40]. They found that those with cognitive impairment in MMSE had significantly higher nighttime systolic BP and more nocturnal BP rise compared to the non-cognitive impairment group. As for the magnetic resonance angiography (MRI) results nocturnal BP rise was significantly associated with greater white matter hyperintensities (WMHs).

#### Hypertension mediated target organ damage

There is, at least, one study which tried to investigate the relationship between systolic Blood pressure variabily [SBPV] and hypertension mediated target organ damage in elderly patients with hypertension. They found the incidences of coronary heart disease and atherosclerotic plaque as well asintima media Thickness (IMT), left ventricular mass Index (LVMI), and microalbuminuria (MA) were higher in the high BPV group than those in the low BPV group (*P* < 0.05 or *P* < 0.01). The multivariate results showed that 24-h SBPV was associated with IMT, LVMI, and MA [41].

#### Heart failure

With increasing age of the population, a high proportion of elderly hypertensives become HF patients, but still prospective studies are required to further investigate the optimal blood pressure target for patients with heart failure [42].

As for heart failure (HF), in the Japan ABPM prospective study, 6359 patients (68.6 ± 11.7 years of age, 48% male patients) with at least one cardiovascular risk factor, without cardiovascular disease underwent ABPM at baseline, and were followed annually for possible atherosclerotic cardiovascular disease and HF. The authors found that nighttime BP levels and a riser pattern were independently associated with the total cardiovascular event rate, for HF [43].

Three studies were aimed to evaluate whether ABPM can identify elderly subjects with HF with a worse prognosis. The University of Nara HF study was aimed to assess if they were any association between outcome of elderly patients with different types of HF and BP measured by ABPM. Nocturnal hypertension and nondipper pattern were not associated with either all-cause or cardiovascular mortality in patients with HF with reduced ejection fraction (HFrEF), HF with mid-range EF (HFmrEF), or HF with preserved EF (HFpEF), except for sleep-time systolic BP in HFrEF. However, the riser pattern was a significant and independent predictor of all-cause and cardiovascular deaths in patients with HFpEF [44].

Komori et al. [43] tested if riser BP pattern is associated with adverse outcomes in HFpEF. And found that those with riser BP pattern subgroup had a significantly higher incidence of the composite outcome than the other subgroups of HFpEF patients [45]. Our group has shown, that in older patients with chronic HF (76.3% with HFpEF), a nondipper or riser BP pattern measured by ABPM was associated with a higher risk of hospitalization and death due to HF [46].

Finally, the ambulatory BP in HFpEF Outcomes Global Registry (HFPEF Global, NCT04065620) is an ongoing observational cohort study that has been designed to assess the association ABPM with cardiovascular outcomes in HFpEF, taking also into considerations confounders such as comorbidities, frailty, and functional capacity [47].

#### Cerebrovascular disease

About cerebrovascular disease in the elderly, the INFINITY trial tried to elucidate the effects of intensive versus standard lowering of BP on ABPM, (measured by 24-h average systolic BP) on mobility, white matter disease progression, and cognitive function after 3 years in elderly hypertensives (age ≥ 75 years) with MRI evidence of WMH lesions. Intensive lowering did not result in differences in mobility outcomes but was associated with a reduction in accrual of subcortical white matter disease [48].

In the Oxford Vascular Study, the association between beat-to-beat BPV and cardiovascular events in elderly patients, 6 weeks after a transient ischemic attack or nondisabling stroke was assessed. Beat-to-beat BPV predicted recurrent stroke and cardiovascular events, independently of mean systolic BP and risk factors [49].

#### Intensive BP control for the elderly

As shown in SPRINT and HYVET, achieved daytime systolic BP of about 126 mmHg was tolerable to the elderly patient during intensive BP control. Because BP measurement in the clinic, measured by automated office blood pressure measurement [AOBPM], and conventional clinic BP can be differed, according to the reference daytime BPs measured by ABPM, masked uncontrolled hypertension or white coat uncontrolled hypertension could be found [50]. In the setting when home BP was not consistent or borderline, ABPM could provide the most accurate achieved BPs during intensive BP control.

## Conclusions

Hypertension management in elderly hypertensive subjects should include a comprehensive evaluation, to detect also comprehensively their cardiovascular risk. Such an evaluation will help us to know whether a BP lowering strategy, could result in a net benefit, for older patients, and do not increase the safety risks, which are associated to these strategies. Those include OH, WCH, falls, and polypharmacy. This evaluation could also include other parameters related to ABPM, as BP variability, nocturnal hypertension or dipper pattern, which have been associated to higher CV risk. Therefore, ABPM could be a useful tool that could answer many of these questions, and to improve hypertension management in older patients. More specific studies investigating the relevance of ABPM in elderly subjects are needed.

## Supplementary Information

**Additional file 1.**

## Data Availability

All data generated or analyzed during this study are included in this published article. For other data, these may be requested through the corresponding author.

## Abbreviations

ABPM: Ambulatory blood pressure measurement
AOBPM: Automated office blood pressure measurement
ARV: Average real variability
BP: Blood pressure
BPV: Blood pressure variability
CV: Cardiovascular
CVD: Cardiovascular disease
EF: Ejection Fraction
HBPM: Home blood pressure measurement
HF: Heart failure
HFmrEF: Heart failure with mid-range EF
HFpEF: Heart failure with preserved EF
HFrEF: Heart failure with reduced EF
HYVET: Hypertension in the very elderly trial
IMT: Intima Media Thickness
LVMI: Left Ventricular Mass Index
MA: Microalbuminuria
MRI: Magnetic resonance angiography
MMSE: Mini-Mental State Examination
OBPM: Office blood pressure measurement
OH: Orthostatic hypotension
WCH: White coat hypertension
WMH: White matter hyperintensities
