# Supplementary material for: Role of ambulatory blood pressure monitoring in elderly hypertensive patients
Source: Clin Hypertens. 2022 Jul 1;28:22. doi: 10.1186/s40885-022-00205-6 (PMC9248111; doi:10.1186/s40885-022-00205-6)
Supplement: Supplementary file 1 — Additional file 1. [file 40885_2022_205_MOESM1_ESM.pdf]

WOLTERS KLUWER HEALTH, INC. ORDER DETAILS

Mar 25, 2022

Order Number	501720451
Order date	Mar 25, 2022
Licensed Content Publisher	Wolters Kluwer Health, Inc.
Licensed Content Publication	Journal of Hypertension
Licensed Content Title	2021 European Society of Hypertension practice guidelines for office and out-of-office blood pressure measurement
Licensed Content Author	George S. Stergiou, Paolo Palatini, Gianfranco Parati, et al
Licensed Content Date	Mar 12, 2021
Licensed Content Volume	39
Licensed Content Issue	7
Type of Use	Journal/Magazine
Requestor type	University/College
Sponsorship	No Sponsorship

Format	Electronic
Portion	Figures/tables/illustrations
Number of figures/tables/illustrations	1
Author of this Wolters Kluwer article	No
Will you be translating?	No
Publishing Open Access	Yes
Creative Commons License	CC-BY
Intend to modify/change the content	Yes
Title of new article	Role of ambulatory blood pressure monitoring in elderly hypertensive patients
Lead author	Miguel Camafort
Title of targeted journal	Clinical Hypertension
Publisher	biomed Central
Expected publication date	May 2022
Order reference number	ClinHYP1
Portions	Table 6 Page 1298

Requestor Location	University of Barcelona<br>Facultad de Medicina<br>Hospital Clínic<br>Casanova 143<br>Barcelona, Barcelona 08036<br>Spain<br>Attn: University of Barcelona
Publisher Tax ID	EU826013006
Total	Not Available
